# Characteristics of Falls Among Men’s Wheelchair Rugby Players in the Rio 2016 and Tokyo 2020 Summer Paralympic Games: A Video Analysis

**DOI:** 10.2478/hukin-2022-0104

**Published:** 2022-11-08

**Authors:** Tsubasa Tashiro, Noriaki Maeda, Junpei Sasadai, Reia Shimizu, Akira Suzuki, Makoto Komiya, Kazuki Fukui, Shogo Tsutsumi, Satoshi Arima, Kazuki Kaneda, Mitsuhiro Yoshimi, Rami Mizuta, Takeru Abekura, Hinata Esaki, Tomoki Terada, Yukio Urabe

**Affiliations:** 1 Graduate School of Biomedical and Health Sciences, Hiroshima University, 1-2-3 Kasumi, Minami-ku, Hiroshima, 734-8553, Japan; 2 Sports Medical Center, Japan Institute of Sports Sciences (JISS), 3-15-1 Nishigaoka Kita-ku, Tokyo, 115-0056, Japan

**Keywords:** para-sports, fall frequency, functional classification, team sports

## Abstract

Wheelchair rugby is a contact sport in which falls are common and injury rates are high, yet the characteristics of the falls are still under-reported. We investigated the fall characteristics of men’s wheelchair rugby players by functional classification, using all 36 official match videos from the Rio 2016 and Tokyo 2020 summer Paralympic Games. The videos were analyzed to evaluate the number of falls, playing time when the fall occurred, playing phase (offense or defense), contact with other players, foul judgement, direction of the fall, and the body part first in contact with the floor. All 182 men’s wheelchair rugby players (Rio 2016, 94; Tokyo 2020, 88) were classified as low-point players or high-point players depending on their functional classification. A total of 200 falls were detected, 27 (13.5%) for low-point players and 173 (86.5%) for high-point players. Significant differences were noted between low-point players and high-point players in the direction of the fall and body part first in contact with the floor. High-point players had more falls in the forward and left-right directions, whereas low-point players were characterized by a higher percentage of falls in the left-right and backward directions. Additionally, high-point players landed on the floor with their hands with high frequency, whereas low-point players landed with their elbows and shoulders more often. Our findings suggest the significance of devising measures to prevent falls during men’s wheelchair rugby games according to their functional classification.

## Introduction

Wheelchair rugby (WR), a paralympic discipline, is a contact sport that includes intentional collisions between wheelchairs which can increase the sport-related injury risk (Vanlandewijck et al., 2001). According to the epidemiological study of the injuries associated with the London 2012 Paralympic Games, the rate of acute injuries in WR (61%) was the second highest among wheelchair sports following that of wheelchair basketball (65%) (Willick et al., 2013). In WR, falls occur as a result of intense contact, which can lead to upper extremity injuries as well as serious head trauma, such as a concussion. Nevertheless, it has been reported that there is a lack of prescribed measures to prevent injuries of wheelchair athletes at the Paralympic Games (Webborn et al., 2017). Adequate knowledge of WR-related injuries can aid coaches, trainers, and athletes to minimize the occurrence of such injuries (Molik et al., 2013).

Injuries resulting from sudden falls during WR, and their mechanism has been clarified in few studies which further explained the characteristics of the direction and situation of such falls. One of the ways to analyze the mechanism of such injuries associated with wheelchair sports is to analyze the falls from in-game videos. A previous video-based study of wheelchair sports reported that, in WR, there were more lateral falls compared to those in wheelchair basketball (Sasadai et al., 2020). However, that study examined the characteristics of falls without accounting for the functional classification (seven classes from 0.5 to 3.5). Since the types and mechanisms of injury may differ among functional classes in WR, understanding the fall characteristics of each class is essential for devising preventive measures against fall-related injuries.

Therefore, the purpose of this study was to investigate the characteristics of falls during games in different functional classes by analyzing videos of games of men’s WR players who participated in the Rio 2016 and Tokyo 2020 Paralympic Games.

## Methods

### Design and Procedures

We conducted a retrospective video analysis study to investigate the characteristics of falls in male WR players by functional classes. All 36 official match videos from the national WR teams that entered in the Rio 2016 and Tokyo 2020 summer Paralympic Games were obtained from the International Paralympic Committee’s (IPC) official website channel of YouTube (https://www.youtube.com/c/paralympics) Eight countries were registered for each of the WR competitions at the Rio 2016 and Tokyo 2020 summer Paralympic Games.

The present study was approved by the Epidemiology Ethics Committee (approval ID, E-1459) and was conducted with no participants’ involvement. Since our video analysis used data that had already been recorded, participants were not involved in the methodology or the interpretation of the results. In addition, participants’ opinions were not reflected in the preparation of this manuscript. Informed consent was waived due to the retrospective nature of the study. This study was conducted with the permission of the IPC.

### Participants / Study sample

In this study, 88 male WR athletes who took part in the Rio 2016 and 94 who participated in the Tokyo 2020 summer Paralympic Games were included in the analysis (Table 1). Data regarding players’ characteristics (age, gender, functional classification) were obtained from the IPC website. Based on their functional classification, WR athletes were classified as low-point players (LPPs; ≤1.5) and high-point players (HPPs; 2.0 or more) (Bauerfeind et al., 2015).

**Table 1:** Demographic profile of men’s wheelchair rugby players who participated in the Rio 2016 and Tokyo 2020 Paralympic Games.

	In Rio 2016 (n = 94)	In Tokyo 2020 (n = 88)
Age (mean ± SD)	32.4 ± 6.2	34.1 ± 6.4
Functional classification, n(%)		
0.5	14(14.9)	12(13.6)
1	14(14.9)	17(19.3)
1.5	8(8.5)	8(9.1)
2	22(23.4)	18(20.5)
2.5	14(14.9)	6(6.8)
3	15(16.0)	18(20.5)
3.5	7(7.4)	9(10.2)

### Measures

A WR game consists of four 8-min periods. Three physical therapists who have been working as sports trainers with para-sports teams for more than three years watched the video and analyzed the characteristics of falls during the game. Each physical therapist watched all the videos in triplicate. They viewed the videos repeatedly as needed and viewed the sequences at normal speed, slow speed, or as still images. The number of falls, playing time at the fall, playing phase (offense or defense), contact with other players, foul judgement, direction of the fall, and the body part first in contact with the floor were recorded. A fall was defined as contact of any body part with the floor (Sasadai et al., 2020). As training before conducting the present analysis, we conducted a pilot analysis of the same match video with three examiners. All examiners were trained to analyze the match with the same criteria regarding the falls.

### Statistical Analysis

SPSS statistics version 27.0 (IBM japan, Tokyo, Japan) was used to conduct all statistical analyses. The Pearson’s X2 test or the Fisher’s exact test was used to compare each categorical variable between the two groups of LPPs and HPPs; the Fisher’s exact test was conducted when the expected number was <5. The level of significance was 5%. The results of categorical variables were reported in agreement with the ratings of two of the three observers. When two or more observers agreed with all category items and the kappa coefficient was >0.8, there was a strong agreement across the three observers on all variables.

## Results

Table 2 shows the characteristics of the fall situation by functional classification. A total of 200 falls were recorded, 27 (13.5%) for LPPs and 173 (86.5%) for HPPs, with a mean of 0.75 and 4.81 falls per match, respectively. Significant differences were found between LPPs and HPPs in the direction of the fall (*p* = 0.027) and the body part first in contact with the floor (*p* = 0.023). No significant differences were observed among LPPs and HPPs in playing time, playing phase, or contact with other players.

**Table 2:** Fall situation characteristics by classification.

	Falls in LPPs (n = 27)	Falls in HPPs (n = 173)	*p*
Playing time (%)			0.287
First quarter	7(25.9)	42(24.3)	
Second quarter	6(22.2)	35(20.2)	
Third quarter	8(29.6)	44(25.4)	
Fourth quarter	6(22.2)	47(27.2)	
Over time	0(0.0)	5(2.9)	
Playing phase (%)			0.274
Offence	13(48.1)	100(57.8)	
Defense	14(51.9)	71(41.0)	
Unidentified	0(0.0)	2(1.2)	
Contact with another player (%)			0.167
Contact	25(92.6)	152(87.9)	
Non-Contact	1(3.7)	18(10.4)	
Unidentified	1(3.7)	3(1.7)	
Foul judgement (%)			0.916
No	19(70.4)	120(69.4)	
Foul	8(29.6)	53(30.6)	
Direction of the fall (%)			0.027
Forward	2(7.4)	52(30.1)	
Backward	5(18.5)	22(12.7)	
Left	11(40.7)	50(28.9)	
Right	8(29.6)	43(24.9)	
Unidentified	1(3.7)	6(3.5)	
Body part first in contact with the floor (%)			0.023
Hand	11(40.7)	118(68.2)	
Elbow	7(25.9)	31(17.9)	
Shoulder	6(22.2)	8(4.6)	
Back	2(7.4)	10(5.8)	
Unidentified/combined	1(3.7)	6(3.5)	

*LPPs, low-point players; HPPs, high-point players; (%) represents the ratio of each item to the total number of falls for LPPs and HPPs, respectively*.

## Discussion

This study aimed to examine fall characteristics during games in different functional classes by analyzing match videos of men’s WR players in the Rio 2016 and Tokyo 2020 Paralympic Games. Of the 200 falls in this study, 173 occurred in HPPs, who may have had a higher percentage of falls than LPPs due to their relatively mild level of physical impairment and their aggressive role. The main findings of our study were in the direction of the fall and the body part first in contact with the floor. The most common fall in HPPs was to the forward direction (30.1%), followed by left (28.9%) and right (24.9%) falls. Interestingly, left (40.7%), right (29.6%), and backward (18.5%) falls were more frequent in LPPs, and the trend of the fall direction differed from that in HPPs. Regarding the body part first in contact with the floor, the percentage of cases with the hand being first in contact with the floor (68.2%) was greater in HPPs. However, the percentages for the elbow (25.9%), shoulder (22.2%), and back (7.4%) were all higher in LPPs than in HPPs. Our findings suggest the need to implement different fall prevention measures for each class.

One reason for the difference in the characteristics of the direction of the fall between LPPs and HPPs would be the speed of the wheelchair movement at the time of the fall. Sporner et al. (2009) reported that the higher the class of a WR athlete, the faster the movement speed. HPPs have a higher wheelchair peak velocity due to better overall muscle strength compared to LPPs (Bakatchina et al., 2021). Moreover, they move around the court quickly for offense and defense and actively engage in ball contests with contact. Therefore, falls in HPPs due to contact at high speed were observed, and there were several instances of falling with the hands in the forward and left-right directions. It is logical that falls to the forward, left, and right would be more frequent because of the greater forward inertia when crashing into a player in front at high speed. This result is supported by a report concluding that HPPs have higher peak velocities and cycle frequencies than LPPs and that HPPs have a higher risk of upper limb injury from falls (Bakatchina et al., 2021). In contrast, LPPs have lower muscle strength in the upper body and are unable to produce superior wheel velocity (Haydon et al., 2018). Thus, their speed of the wheelchair movement is slower than that of HPPs. This fact may explain why LPPs are contacted more often by HPPs, causing them to fall unexpectedly to the left, right, or backward. Even if LPPs are contacted, tackling the vehicle rearward of the rear axle is a "spinning foul", and thus may inevitably result in fewer forward falls. In addition, LPPs have poor wrist function and may not be able to land on their hands during a fall, increasing the risk of concussion. Given that fewer forward falls occurred and that trunk and upper extremity function was weaker than in HPPs, the inability to control the body so that the hands land on the floor during a fall may reflect these results. For injury prevention measures related to falls in WR players, it should be considered to instruct HPPs to absorb impact with their hands in the forward falls and to wear helmets as well as elbow and shoulder protector pads for LPPs, based on the results of our study.

Moreover, the shape of the wheelchair may explain the fall characteristics of different functional classes. Offensive wheelchairs, used by players with less severe disabilities, have a higher seat surface, whereas defensive wheelchairs, used by players with more severe disabilities, have a lower seat surface and are angled so that the front of the seat is higher. As this difference affects the height of the players’ center of gravity, HPPs, who often use wheelchairs with a higher seat surface, fell more frequently than LPPs during games. Also, the front bumper of the LPP is often longer than that of the HPP, making it less likely to fall forward. Future research should investigate the speed during falls and the wheelchair shape in WR matches to reveal additional details on the characteristics of falls by functional class.

This study had several limitations. First, only official IPC videos were analyzed; hence, it was difficult to record falls that were not officially recorded. Second, it was not possible to detect the speed of the wheelchair movement at the time of the fall from the video. Last, we were unable to identify any injuries resulting from falls during games. However, this study provides important insight into the profile of fall-related injuries (e.g., concussions) in Paralympic sports. Additional research is required to determine the relationship between falls and injury occurrence in male WR athletes.

## Conclusion

The videos of all 36 Rio 2016 and Tokyo 2020 summer Paralympic Games were analyzed to examine the characteristics of falls during the games among men’s WR players. The direction of the fall and the body part first in contact with the floor tended to differ by the functional level. Coaches and trainers involved in WR may be required to take preventive measures against the occurrence of fall-related injuries at different levels of functional classification.
